# Co-infections in Persons with Early Lyme Disease, New York, USA 

**DOI:** 10.3201/eid2504.181509

**Published:** 2019-04

**Authors:** Gary P. Wormser, Donna McKenna, Carol Scavarda, Denise Cooper, Marc Y. El Khoury, John Nowakowski, Praveen Sudhindra, Alexander Ladenheim, Guiqing Wang, Carol L. Karmen, Valerie Demarest, Alan P. Dupuis, Susan J. Wong

**Affiliations:** New York Medical College, Valhalla, New York, USA (G.P. Wormser, D. McKenna, C. Scavarda, D. Cooper, M.Y. El Khoury, J. Nowakowski, P. Sudhindra, A. Ladenheim, G. Wang, C.L. Karmen);; New York State Department of Health, Albany, New York, USA (V. Demarest, A.P. Dupuis II, S.J. Wong)

**Keywords:** Lyme disease, co-infection, Anaplasma, Babesia, Borrelia miyamotoi, Borrelia burgdorferi, erythema migrans, Powassan virus, bacteria, parasites, viruses, New York, United States

## Abstract

In certain regions of New York state, USA, *Ixodes scapularis* ticks can potentially transmit 4 pathogens in addition to *Borrelia burgdorferi*: *Anaplasma phagocytophilum*, *Babesia microti*, *Borrelia miyamotoi*, and the deer tick virus subtype of Powassan virus. In a prospective study, we systematically evaluated 52 adult patients with erythema migrans, the most common clinical manifestation of *B. burgdorferi* infection (Lyme disease), who had not received treatment for Lyme disease. We used serologic testing to evaluate these patients for evidence of co-infection with any of the 4 other tickborne pathogens. Evidence of co-infection was found for *B. microti* only; 4–6 patients were co-infected with *Babesia microti*. Nearly 90% of the patients evaluated had no evidence of co-infection. Our finding of *B. microti* co-infection documents the increasing clinical relevance of this emerging infection.

Lyme disease, caused by *Borrelia burgdorferi*, is the most common tickborne infection in North America (*1*,*2*). The Lower Hudson Valley, in the US state of New York, is a region of high risk for bites from *Ixodes scapularis* ticks (*3*). *I. scapularis* ticks in this region, and in certain other geographic areas in New York and the northeastern United States, are responsible for transmission of 4 other pathogens besides *B. burgdorferi*: *Anaplasma phagocytophilum*, the cause of human granulocytic anaplasmosis; *Babesia microti*; *Borrelia miyamotoi*; and the deer tick virus subtype of Powassan virus (POWV) (*4*–*9*). The earliest and most common clinical manifestation of Lyme disease is the lesion erythema migrans. To look for evidence of co-infection with these 4 tickborne pathogens, we tested the serum of 52 adult patients with erythema migrans who had not received treatment for Lyme disease.

## Methods

### Patient Cohort

We enrolled adult patients with Lyme disease in a prospective study at the Lyme Disease Diagnostic Center, in Westchester County in the Lower Hudson Valley region of New York, to assess the outcome of this infection over 1 year, as described elsewhere (*10*). The Lower Hudson Valley region of New York is defined as Westchester, Putnam, Dutchess, Rockland, Orange, Ulster, and Sullivan Counties. This report focuses on 52 persons who at the time of study entry had erythema migrans but no treatment for Lyme disease and no clinical evidence of a concomitant extracutaneous manifestation of Lyme disease. Each patient had >1 expanding erythematous skin lesion that was >5 cm in diameter (*11*,*12*). 

For each of the 52 persons with erythema migrans, baseline visits occurred from June 2, 2011, through July 30, 2015; blood was collected before antimicrobial drug treatment began (baseline) and at the next follow-up visit. The study was approved by the institutional review board at New York Medical College.

### Testing and Confirmation of Infection

Serologic testing to document co-infection was performed retrospectively. Testing for antibodies to *A. phagocytophilum* was conducted by immunofluorescent assay at Focus Diagnostics, Inc. (Cypress, CA, USA) (*10*). Testing for antibodies to *B. microti* was done by immunofluorescent assay at either Focus Diagnostics, Inc., or at the New York State Department of Health Wadsworth Center (Albany, NY, USA) (*10*).

Testing for antibodies to the glycerophosphodiester phosphodiesterase (GlpQ) protein of *B. miyamotoi* was performed at the Wadsworth Center by using a microsphere immunoassay that detects total antibodies (IgG + IgA + IgM) to recombinant GlpQ of *B. miyamotoi*. The recombinant GlpQ was kindly provided by Sukanya Narasimhan and Erol Fikrig of Yale University (New Haven, CT, USA).

Testing for antibodies to POWV was also performed at the Wadsworth Center (*8*). Serologic testing for POWV infection included a microsphere immunoassay to detect total antibodies (IgG + IgA + IgM) to recombinant deer tick virus envelope protein and an IgM capture enzyme immunoassay to the LB strain of POWV (*8*). When the microsphere immunoassay and the IgM capture enzyme immunoassay were both reactive on acute- or convalescent-phase serum specimens, plaque reduction testing for neutralization antibodies against the LB strain of POWV was also performed. 

Serologic evidence of *A. phagocytophilum* co-infection required a 4-fold rise in IgG titer between the acute- and convalescent-phase samples to >1:512 (*13*). Serologic evidence of *B. microti* infection required a 4-fold rise in IgG titers between the acute- and convalescent-phase serum samples. Patients who had clinical evidence of *A. phagocytophilum* or *B. microti* infections (e.g., new onset of fever, characteristic hematologic abnormalities, or both), or in whom such clinical evidence developed, were also tested when these findings were observed, by blood smear, PCR, or both, to detect these microorganisms, as described elsewhere (*13*,*14*). Because a positive result on the acute-phase sample is atypical for acute *B. miyamotoi* infection, serologic evidence of *B. miyamotoi* infection required seroconversion in the convalescent-phase sample for antibody to GlpQ (*15*). A diagnosis of possible POWV co-infection required a finding of positive IgM and neutralizing antibodies to POWV for the same serum sample. A confirmed diagnosis of POWV co-infection, however, required a 4-fold rise in neutralizing antibodies between the acute- and convalescent-phase serum samples; in addition, the neutralizing titer value had to exceed by >4-fold the neutralizing antibody titers found by using the patient’s serum under the same test conditions but using other flaviviruses, such as West Nile virus.

### Other Assessments

At each patient’s baseline visit, we collected demographic and clinical data, including data on 12 somatic symptoms (e.g., fatigue, headache, joint pain, muscle pain, and cognitive complaints), as described elsewhere (*10*,*16*). We performed serologic testing for Lyme disease at both the baseline and convalescent visits by using the C6 Lyme ELISA kit (Immunetics, Inc., http://www.oxfordimmunotec.com) according to the manufacturer’s recommendations. 

### Statistical Methods

For comparison of categorical variables, we used the Fisher exact test or the exact McNemar test; for continuous variables, we used the Student *t*-test. All testing was 2-tailed. We considered p<0.05 to be significant.

## Results

At the time of the baseline visit, none of the 52 patients with erythema migrans had received antimicrobial drugs; 31 (59.6%) patients had 1 erythema migrans lesion, and 21 (40.4%) patients had multiple erythema migrans lesions (Table 1). Thirty-four (65.4%) patients were male, mean age ± SD was 50.2 ± 15.7 years (range 20–86 years), and 39 (75.0%) patients had concomitant subjective symptoms such as fatigue. All but 4 patients had been exposed to ticks while in areas that included the Lower Hudson Valley (Table 1).

**Table 1:** Demographics and sites of potential tick exposure for 52 participants in study of co-infections in persons with Lyme disease, New York, USA, June 2, 2011, through July 30, 2015*

Variable	No. (%)
Sex	
M	34 (65.4)
F	18 (34.6)
Multiple erythema migrans skin lesions	21 (40.4)
Tick exposure	
Potential exposure in at least LHV	48 (92.3)
Potential tick exposure in LHV alone	32 (61.5)
No tick exposure in LHV†	4 (7.7)

*Mean age 50.2 ± 15.7 y, range 20–86 y. LHV, Lower Hudson Valley of New York state, USA (includes Westchester, Putnam, Dutchess, Rockland, Orange, Ulster, and Sullivan Counties). †Two participants were exposed to ticks in Long Island, New York, and 2 in Connecticut.

Convalescent-phase blood samples, which were obtained at a mean of 16.7 days (range 7–30 days) after the baseline visit, were screened for antibodies to the C6 peptide of *B. burgdorferi* and for antibodies to *A. phagocytophilum*, *B. microti*, GlpQ protein of *B. miyamotoi*, and POWV. A total of 46 (88.5%) patients were seropositive by the C6 Lyme ELISA, 32 (69.6%) on both the baseline and convalescent-phase blood samples, and 14 (30.4%) on the convalescent-phase sample only.

None of the 52 patients had evidence of *A. phagocytophilum* co-infection, although 4 had an IgG titer of 1:64 on the convalescent-phase blood sample, which is regarded as a nonspecific finding (*13*,*17*,*18*). Titers <1:64 were considered to be negative by the performing laboratory and thus were not reported. 

Of the 52 patients, 4 (7.7%, 95% CI 3%–18%) had convincing evidence of *B. microti* co-infection (Table 2), and active babesiosis was clinically suspected for 3 of these patients. For 1 of the 3 patients who had clinical evidence of active babesiosis and a single erythema migrans lesion, fever developed on day 4 of amoxicillin therapy. Another of these patients underwent diagnostic testing for babesiosis because of fever before development of a single erythema migrans lesion. The third patient, who was afebrile, was tested for babesiosis 2 days after beginning antimicrobial drug treatment for a single erythema migrans lesion because of thrombocytopenia and anemia that were documented at the time of study entry. These 3 patients were positive for *B. microti* DNA by PCR, and 1 of the 3 also had a positive blood smear. All 3 patients received a course of treatment for babesiosis.

**Table 2:** Participants with evidence of *Babesia microti* co-infection in study of co-infections in persons with Lyme disease, New York, USA, June 2, 2011, through July 30, 2015*

Age, y	Fever	No. symptoms at baseline visit	Tick exposure in LHV	Tick exposure outside LHV	Baseline *B. microti* antibody titer	Convalescent-phase *B. microti* antibody titer (timing, d)†	Blood smear	PCR for *Babesia* DNA
69	Yes, but began 4 d after baseline visit while taking amoxicillin for treatment of Lyme disease	1	Yes	No	>1:1,024	>1:1,024 (12)	+	+
58	Yes, began 1 or 2 d before the baseline visit	10	Yes	Yes	<1:64	>1:1,024 (14)	–	+
61	No	2	Yes	No	>1:1024	>1:1,024 (21)	–	+
45	No	1	Yes	No	<1:64	1:512 (18)	ND	ND
54	No	2	No	Yes	>1:1024	>1:1,024 (19)	ND	ND
32	No	4	Yes	No	>1:1024	>1:1,024 (14)	ND	ND

*LHV, Lower Hudson Valley of New York state, USA (includes Westchester, Putnam, Dutchess, Rockland, Orange, Ulster, and Sullivan Counties); ND, not done; +, positive; –, negative. †Time from baseline visit.

A fourth patient without a febrile illness had a convalescent-phase IgG titer of 1:512 and an acute-phase titer of <1:64, consistent with co-infection with *B. microti*. In addition, 2 patients without clinical evidence of a febrile illness had acute- and convalescent-phase IgG titers of >1,024 (Table 2). Because the exact titer for these serum specimens was not determined, it was not possible to determine if the convalescent-phase sample demonstrated a 4-fold increase in titer. Although none of these 3 patients received anti-*Babesia* drug therapy, all recovered fully from Lyme disease during the 1-year follow-up period.

Therefore, up to 6 (11.5%; 95% CI 5%–23%) of the 52 patients may have been co-infected with *Babesia*; 3 of these patients were known to have had fever, hematologic findings consistent with active *Babesia* infection, or both. All 6 had >1 nonspecific symptoms at study entry; mean ± SD was 3.3 ± 3.4 symptoms (range 1–10 symptoms). In comparison, the mean number of symptoms for the other 46 patients at the baseline visit was 3.1 ± 3.3 (range 0–12 symptoms; p = 0.88). One additional patient had a convalescent-phase IgG titer of 1:128 and an acute-phase IgG titer of 1:256, possibly indicative of a prior *Babesia* infection antedating the onset of Lyme disease.

None of the 52 patients met criteria for serologic evidence of *B. miyamotoi* co-infection, although 4 (7.7%) were seropositive for antibodies to GlpQ on acute- and convalescent-phase serum samples but without a discernible increase in values on the convalescent-phase sample. In addition, none of the 52 patients met criteria for serologic evidence for possible or confirmed POWV co-infection; 1 serum sample was positive for IgM to the LB strain of POWV but negative for neutralization antibodies to both the LB strain and a deer tick virus subtype strain of POWV. Therefore, more patients had laboratory evidence of co-infection with *Babesia* than with *A. phagocytophilum*, *B. miyamotoi*, or POWV (possibly as high as 6 [11.5%] for *Babesia* vs. 0 for the other pathogens tested; p = 0.031). 

## Discussion

In this study of 52 adult patients who had erythema migrans but had not been treated for Lyme disease, conducted in the Lower Hudson Valley of New York, the only documented *B. burgdorferi* co-infection was with *B. microti*. Several prior studies that used PCR have evaluated *I. scapularis* ticks found in this region for co-infection with *B. burgdorferi*; these studies found values of up to 30% for co-infection with *A. phagocytophilum* (*4*,*5*,*9*,*19*), up to 24% for *B. microti* (*4*,*5*,*9*,*19*), 1% for *B. miyamotoi* (*4*), and up to 3.9% for POWV (*4*,*9*). In general, lower rates of co-infection were associated with *I. scapularis* ticks in the nymphal stage than in the adult stage; this finding is relevant to our study because most cases of early Lyme disease in this region result from bites of ticks in the nymphal stage (*20*).

Extrapolating data on the rate of co-infections by PCR testing of ticks to human co-infection rates should be done cautiously. Confounding factors are the possible existence of nonpathogenic strains of *Anaplasma* or *Babesia* in ticks and whether these organisms may have contributed to a portion of the positive PCR results for *A. phagocytophilum* (*21*) or *B. microti.* For example, *B. odocoilei* is found in *I. scapularis* ticks but is not regarded as a human pathogen (*22*). In addition, the potential tick exposure locations of participants in our study were not restricted to the Lower Hudson Valley region of New York. Indeed, tick exposure for 4 of the 52 study participants occurred exclusively in Long Island or Connecticut (Table 1), and 1 of these 4 participants was among the 6 participants with laboratory evidence of *B. microti* co-infection (Table 2). This participant had no clinical evidence of a febrile illness but had acute- and convalescent-phase *B. microti* IgG titers >1,024.

We systematically evaluated adult patients with erythema migrans for co-infection with 4 *I. scapularis* tick–transmitted pathogens. We used well-defined and highly rigorous criteria for defining co-infection and focused on consecutively enrolled patients with the most certain clinical marker of early Lyme disease; namely, an erythema migrans lesion (*12*). Studies using less stringent case definitions may potentially detect higher numbers of putative co-infections but with less certain validity and less clarity for differentiating sequential from simultaneous infections (*13*,*23*). Unlike a previous study of untreated *Babesia* co-infections in patients considered to have Lyme disease (*24*), patients in our study with evidence of *Babesia* co-infection at baseline evaluation were not more symptomatic than those without this co-infection.

Limitations of our study are the relatively small sample size and the assumption that the convalescent-phase serum samples were obtained at the appropriate time to reliably identify the co-infections assessed (mean time from baseline visit to collection of the convalescent- phase blood sample was 16.7 days [range 7–30 days]). Most (75%) of the 52 patients in our study had received doxycycline, raising the question of whether this treatment may have affected the likelihood of seroconversion for antibodies to *A. phagocytophilum.* However, patients with culture-confirmed human granulocytic anaplasmosis regularly produce high antibody titers within 2 weeks of symptom onset despite receipt of doxycycline (*17*). Thus, the only theoretical concern about whether doxycycline might have reduced the observed frequency of *A. phagocytophilum* co-infection would have been for cases of incubating infection that might have been prevented from becoming active.

Another possibility, however, is that we excluded patients whose fever or systemic symptoms were primarily caused by human granulocytic anaplasmosis, rather than Lyme disease, and thus had started antimicrobial drug therapy before study entry. To address this question, we separately looked at acute- and convalescent-phase antibody titers to *A. phagocytophilum* in 38 patients with erythema migrans who were enrolled into the same study but for whom antimicrobial drug therapy had been initiated before enrollment. For 1 (2.6%) of the 38 patients, we found a 4-fold rise in antibody titers to *A. phagocytophilum* between the acute- and convalescent-phase serum samples. However, this finding did not differ significantly from what we found for the 52 patients with erythema migrans (1/38 vs. 0/52; p = 0.42).

Another study limitation is our use of serologic testing assays that were not approved by the US Food and Drug Administration; consequently, their performance characteristics are uncertain. Last, our results pertain to a particular geographic area over a discrete time frame and may not pertain to other locations or other periods.

In conclusion, we systematically and rigorously evaluated consecutively enrolled adult patients with erythema migrans for co-infection with the 4 other *I. scapularis* tick–transmitted pathogens found in parts of New York and in other geographic areas in the northeastern United States. Nearly 90% of the patients evaluated had no serologic evidence of co-infection. *B. microti* was the only co-infection found, further documenting the clinical relevance of this emerging infection. Similar studies in other geographic areas, in addition to testing acute- and convalescent-phase serum, should include direct diagnostic testing by use of reliable PCR assays to detect potential co-infecting pathogens (particularly for *A. phagocytophilum*, *B. microti*, and *B. miyamotoi*) at the baseline visit.
